# Gene therapy of yeast NDI1 on mitochondrial complex I dysfunction in rotenone-induced Parkinson’s disease models in vitro and vivo

**DOI:** 10.1186/s10020-022-00456-x

**Published:** 2022-03-07

**Authors:** Hongzhi Li, Bohao Sun, Yuting Huang, Jing Zhang, Xuejing Xu, Yuqi Shen, Zhuo Chen, Jifeng Yang, Luxi Shen, Yongwu Hu, Haihua Gu

**Affiliations:** 1 Zhejiang Provincial Key Laboratory of Medical Genetics, Key Laboratory of Laboratory Medicine, Ministry of Education, School of Laboratory Medicine and Life Sciences, Wenzhou Medical University, Chashan University Town, Northern Zhongshan Road, Wenzhou, Zhejiang 325035 People’s Republic of China; 2 Department of Internal Neurology, Beijing Friendship Hospital, Capital Medical University, Beijing, 100050 China

**Keywords:** Parkinson's disease, Rotenone, Yeast NDI1, Mitochondrial complex I, Recombinant adeno-associated virus (rAAV), Gene therapy

## Abstract

**Purpose:**

Parkinson's disease (PD) is the second most common neurodegenerative disease without cure or effective treatment. This study explores whether the yeast internal NADH-quinone oxidoreductase (NDI1) can functionally replace the defective mammalian mitochondrial complex I, which may provide a gene therapy strategy for treating sporadic PD caused by mitochondrial complex I dysfunction.

**Method:**

Recombinant lentivirus expressing NDI1 was transduced into SH-SY5Y cells, or recombinant adeno-associated virus type 5 expressing NDI1 was transduced into the right substantia nigra pars compacta (SNpc) of mouse. PD cell and mouse models were established by rotenone treatment. The therapeutic effects of NDI1 on rotenone-induced PD models in vitro and vivo were assessed in neurobehavior, neuropathology, and mitochondrial functions, by using the apomorphine-induced rotation test, immunohistochemistry, immunofluorescence, western blot, complex I enzyme activity determination, oxygen consumption detection, ATP content determination and ROS measurement.

**Results:**

NDI1 was expressed and localized in mitochondria in SH-SY5Y cells. NDI1 resisted rotenone-induced changes in cell morphology, loss of cell viability, accumulation of *α*-synuclein and pS129 *α*-synuclein, mitochondrial ROS production and mitochondria-mediated apoptosis. The basal and maximal oxygen consumption, mitochondrial coupling efficiency, basal and oligomycin-sensitive ATP and complex I activity in cell model were significantly increased in rotenone + NDI1 group compared to rotenone + vector group. NDI1 was efficiently expressed in dopaminergic neurons in the right SNpc without obvious adverse effects. The rotation number to the right side (NDI1-treated side) was significantly increased compared to that to the left side (untreated side) in mouse model. The number of viable dopaminergic neurons, the expression of tyrosine hydroxylase, total and maximal oxygen consumption, mitochondrial coupling efficiency and complex I enzyme activity in right substantia nigra, and the content of dopamine in right striatum were significantly increased in rotenone + NDI1 group compared to rotenone + vector group.

**Conclusion:**

Yeast NDI1 can rescue the defect of oxidative phosphorylation in rotenone-induced PD cell and mouse models, and ameliorate neurobehavioral and neuropathological damages. The results may provide a basis for the yeast NDI1 gene therapy of sporadic PD caused by mitochondrial complex I dysfunction.

## Introduction

Mammalian mitochondrial respiratory chain complex I (NADH dehydrogenase complex) contains 7 subunits encoded by mitochondrial DNA (mtDNA) and 38 subunits encoded by nuclear DNA. It plays key roles in mitochondrial electron transport compared with other complexes (Lenaz and Genova 2010). Mitochondrial respiratory chain complex I deficiency can lead to diseases in nervous, muscular and other systems including Parkinson's disease (PD), Alzheimer's disease, Leber hereditary optic neuropathy, mitochondrial myopathy and cardiomyopathy (Tzen et al. 2007; Gasparre et al. 2008; Rodenburg 2016).

PD, the second most common neurodegenerative disease affecting 1% of people over 65 years, is clinically characterized by resting tremor, bradykinesia, ankylosis, and abnormal posture balance. PD is caused by genetic and environmental factors, and can be divided into familial (5% of cases) and sporadic (95% of cases) types. According to the mitochondrial dysfunction hypothesis, the pathogenesis of PD (especially sporadic type) is caused by the decrease in complex I activity of the mitochondrial respiratory chain (Bose and Beal 2016; Choong and Mochizuki 2017; Moore et al. 2005). The decrease of complex I activity in sporadic PD patients, obviously, can not be explained all by mutations of the genes encoding the complex I subunits (Kösel et al. 1999; Hattori et al. 1998). It is suggested that the strategy for the treatment of sporadic PD should be rescuing the functional deficiency of the whole complex I, rather than respectively aiming at mutations of the genes encoding complex I subunits.

Current drug therapy for PD using the dopamine precursor l-dopa or dopamine receptor agonists can only relieve symptoms of the disease temporarily but do not stop the progression of the disease or cure the decease. Potential molecular and cellular therapies have been tested for the treatment of PD, including stem cell transplantation therapy with embryonic stem (ES) cells and induced pluripotent stem (iPS) cells (Ge et al. 2018; Muñoz et al. 2019), immunotherapy with antibodies, and gene therapy using viral vector-mediated gene delivery, RNA interference, CRISPR-Cas9 gene editing, and et al. (Jamebozorgi et al. 2019; Lin et al. 2017). Gene therapy for PD offers a promising strategy (Choong and Mochizuki 2017), including delivery of genes encoding proteins that can protect dopaminergic neurons from damage, such as neurotropic factors, anti-apoptotic and anti-free radical proteins (Lin et al. 2017; Valdés and Schneider 2016; Nam et al. 2021), and delivery of genes encoding enzymes involving in dopamine synthesis, for example, aromatic L-amino acid decarboxylase (AADC), tyrosine hydroxylase (TH), GTP cyclohydrolase (GCH) (Jamebozorgi et al. 2019; Lin et al. 2017; Christine et al. 2019; Ciesielska et al. 2017). To date, gene therapy for PD has shown encouraging results in preclinical animal models, but few are being investigated in clinical trials. Phase I and phase II clinical trials of gene therapy for PD investigated or completed in the United States included AAV2-GAD (LeWitt et al. 2011; Niethammer et al. 2017; Kaplitt et al. 2007), AAV2-AADC (Christine et al. 2019, 2009; Mittermeyer et al. 2012; Muramatsu et al. 2010), and AAV2-NRTN (Bartus et al. 2013; Marks et al. 2016, 2010). However, the clinical efficacy remains to be determined (Blits and Petry 2016). Each of the reported PD gene therapy can only be used for a small number of PD patients with specific defect.

Previous studies demonstrated that the yeast complex I (NADH dehydrogenase), composed of a single subunit NDI1(internal NADH-quinone oxidoreductase), could homologously replace the mammalian complex I, composed of 45 subunits (Santidrian et al. 2013; Bordt et al. 2017). We hypothesize that yeast NDI1 could rescue the functional defects of mammalian complex I by homologous replacement.

Studies have demonstrated that long-term exposure to low dosage of rotenone, a pesticide and fish pond cleaning agent, might be the environmental factor contributing to the onset of PD. Rotenone treatment can induce two main characteristics of PD, including selective degeneration of dopaminergic neurons and formation of Lewy bodies in the cytoplasm in cell model (Zhang et al. 2016). Rotenone accelerated the aggregation of *α*-synuclein from monomer to fiber oligomer (Silva et al. 2013). In fact, studies have also shown that mouse treated with rotenone is an ideal animal model for sporadic PD with complex I deficiency, displaying behavioral, pathological, and biochemical characteristics of sporadic PD (Alam and Schmidt 2002; Sherer et al. 2003; Johnson and Bobrovskaya 2015). Rotenone is a specific inhibitor of mammalian complex I, whereas yeast NDI1 is a rotenone-resistant NADH dehydrogenase (Melo et al. 2004).

In this study, SH-SY5Y, a human cell line with neuronal phenotype, was treated by rotenone to establish the PD cell model. We demonstrated the therapeutic effect of yeast NDI1 protein on the defective functionality of complex I and cytopathological features. Furthermore, PD mouse model was established by oral gavage administration of rotenone. We achieved therapeutic impact of yeast NDI1 on mitochondrial function, pathological features, and neuropathy. This study may provide a basis for yeast NDI1 gene therapy for mitochondrial diseases caused by complex I defects, such as sporadic PD.

## Materials and methods

### Cell lines and cell culture

SH-SY5Y cells and 293T-17 cells were purchased from American Type Culture Collection (ATCC, Manassas, VA, USA) and cultured in Dulbecco's modified Eagle's medium (DMEM, Gibco, California, USA) with 10% fetal bovine serum (FBS, Gibco).

### Animals

C57B/L6 male mice (12 weeks old) were purchased from Shanghai Shrek Experimental Animal Co, Ltd. The animal research was approved by the Institutional Animal Care and Use Committee of Wenzhou Medical University.

### Recombinant lentivirus and recombinant adeno-associated virus production

The lentiviral vector pLVX-CMV-HA-NDI1-IRES-ZsGreen1 and adeno-associated viral vector pAAV-CMV-HA-NDI1 expressing NDI1 were constructed by inserting the HA-tag behind the mitochondrial targeting sequence at the N-terminus of NDI1. The recombinant lentiviral plasmid was co-transfected into 293T-17 cells with the packaging plasmids pSPAX2 and pMD2.G using PEI to produce recombinant NDI1 lentivirus (LV-NDI1). The LV-NDI1 supernatant was concentrated and purified using a method previously established by our research group (Yuan et al. 2018). The titer of the concentrated LV-NDI1 typically reached 1 × 10^8^ TU/mL. Recombinant NDI1 adeno-associated virus (AAV-NDI1, AAV5 serotype, titer 2.1 × 10^12^ vg/mL) was packaged and titered by BrainVTA (Wuhan, China).

### Treatment of SH-SY5Y cells with rotenone and recombinant lentivirus

The LV-NDI1 was transduced to human neuronal cells SH-SY5Y at the MOI of 5 in the presence of 8 μg/mL polybrene. For DMSO + vector group (control group) or rotenone + vector group (rotenone model group) or rotenone + NDI1 group (NDI1 treatment group), SH-SY5Y cells were transduced with LV-vector or LV-NDI1, 72 h later treated with DMSO or 1 μM rotenone (Sigma, St Louis, MO, USA) for additional 24 h. The experiments were repeated for three times.

### Establishment of rotenone-induced mouse model and stereotactic injection of recombinant adeno-associated virus (AAV)

Mice were anesthetized with 1.5% isoflurane inhalation and placed in a stereotaxic apparatus (Stoelting, Wood Dale, IL, USA). The AAV5-NDI1 or AAV5-vector was injected using a glass micropipette (diameter 1 mm, tip 5 μm) connected to a 5 μL Hamilton microsyringe. A single injection of 3 µL AAV5-NDI1 or AAV5-vector was delivered to the right cerebral hemisphere at the following substantia nigra coordinates (measured from bregma): AP: − 3.3 mm, ML: 1.5 mm, and DL: − 3.9 mm, at a rate of 0.6 µL/min. Five weeks after rAAV5 injection, mice were gavaged once a day with 30 mg/kg rotenone, in 3 mg/mL suspended in 0.5% sodium carboxymethyl cellulose (CMC, Sigma), for six consecutive weeks. Mice were randomly divided into four groups, including CMC + vector group (control group), CMC + NDI1 group (NDI1 alone group), rotenone + vector group (rotenone model group), and rotenone + NDI1 group (NDI1 treatment group). The experiments were repeated for three times.

### Cell viability assay

Cell viability was analyzed by trypan blue exclusion staining. All the culture supernatant, cell wash solution, and cell suspension after trypsin digestion were collected and centrifuged at 1000 rpm for 3 min. The cell precipitate was suspended in PBS. The cell suspension was mixed well with 0.4% trypan blue (ThermoFisher Scientific, Shanghai, China) at 9:1, and the number of blue cells and total cells were counted immediately under the microscope. Cell viability (%) = [1 − (blue cells/total cells)] × 100.

### Apomorphine-induced rotation assay

Five mice in each group were respectively placed in a cylinder (11.5 cm in diameter × 14 cm in height) in a quiet room. The mice were acclimated to their surroundings for 5 min before being intraperitoneally injected with 0.5 mg/kg apomorphine (Wako Pure Chemical Industries, Osaka, Japan), in 1.25 mg/mL dissolved in physiological saline. Each mouse was monitored for 20 min, and the rotation number to the left or the right of mice was recorded.

### Hematoxylin–eosin (H&E) staining and immunohistochemistry

Five mice in each group were anesthetized with 1.5% isoflurane inhalation and transcardially perfused with 4% paraformaldehyde. Brain tissues encompassing the substantia nigra (SN) and striatum were coronally dissected using mouse brain mold (RWD Life Science, Shenzhen, China) and fixed in 4% formaldehyde solution overnight at 4 ℃. After dehydration, the brains were embedded in paraffin and cut into 4 μm thick coronal sections.

H&E staining of SN tissue sections was by using the hematoxylin–eosin kit (Solarbio, Beijing, China).

For immunohistochemistry (IHC): the tissue sections were boiled in 10 mM citrate buffer (pH 6.0) for antigenic retrieval, and incubated with 3% H_2_O_2_ for 15 min to block endogenous peroxidase activity. The sections were subsequently blocked with 5% goat serum (Phygene, Fuzhou, China) for 45 min, and incubated with primary antibodies including mouse anti-HA antibody (1:200; cat. no. 3724; Cell Signaling Technology, Danvers, MA, USA), rabbit anti-TH antibody (1:1000; cat. No. 22941; Immunostar, Hudson, WI, USA), rabbit anti-pS129 α-synuclein antibody (1:5000; cat. no. ab51253; Abcam, Cambridge, UK) and rabbit anti-glial fibrillary acidic protein (GFAP) antibody (1:1,000; cat. no. ab68428; Abcam, Cambridge, UK) overnight at 4 °C, and subsequently with secondary antibodies including horseradish peroxidase (HRP) conjugated goat anti-mouse IgG antibody (1:1,000; cat. no. ab6823; Abcam) or goat anti-rabbit IgG antibody (1:1000; cat. no. ab6112; Abcam) for 90 min at room temperature. Finally, the sections were developed using a DAB substrate kit (Abcam), and counterstained with hematoxylin (Solarbio, Beijing). Image acquisition was performed using a light microscope (Nikon E200, Tokyo, Japan). TH-positive (brownish yellow) cells in the substantia nigra pars compacta (SNpc) or ventral tegmental area (VTA) and TH-positive or GFAP-positive optical density values in the striatum were quantified using ImageJ software. One field was randomly taken from each of the five different sections in each group, and the average value was calculated.

### Immunofluorescent staining

Cultured cells were seeded on coverslips in 24-well plates and incubated with 200 μL 500 nM Mito-Tracker Red CMXRos (ThermoFisher Scientific) for 30 min at 37 ℃. Then the cells were fixed with 4% paraformaldehyde for 30 min, permeabilized with 0.5% TritonX-100 for 10 min, and blocked with 3% BSA + 0.1% Tween in PBS for 1 h. The samples were incubated with primary anti-HA antibody (1:500; cat. no. 3724; Cell Signaling Technology) overnight at 4 ℃, then with secondary antibodies including Alexa Fluo555-labeled donkey anti-rabbit IgG antibody (1:500; Beyotime Institute of Biotechnology, Shanghai, China) or Alexa Fluo647-labeled goat anti-mouse IgG antibody (1:500; Beyotime Institute of Biotechnology) for 1 h, and followed by DAPI staining for 3 min. Image acquisition was performed using a laser scanning confocal microscope (Nikon A1).

For mouse tissues (five mice in each group), SN tissue sections were boiled in 10 mM citrate buffer (pH 6.0) for antigenic retrieval, and permeabilized with 0.5% TritonX-100 for 20 min. Then the sections were blocked with 5% BSA for 45 min, and incubated with primary antibodies including mouse anti-mouse HA antibody (1:500; cat. no. 3724; Cell Signaling Technology) or rabbit anti-mouse TH antibody (1:500; cat. no. AF2185; Beyotime Institute of Biotechnology) overnight at 4 ℃. The following steps were the same as the immunofluorescent staining protocol for cultured cells.

### Western blot

Mice were anesthetized with 1.5% isoflurane inhalation. Fresh brain tissues encompassing SN and striatum were coronally dissected using mouse brain mold (after then the mice were euthanized), then fresh SN or striatum tissue was dissected according to mouse brain map. Cells, or fresh tissues from mice (4 per group), were homogenated in RIPA lysis buffer containing PMSF protease inhibitor with a tissue homogenizer, then incubated on ice to lyse for 30 min, and centrifuged at 12,000*g* for 20 min at 4 ℃. The protein concentration of the supernatants (cell or tissue lysates) was determined using BCA kit. The supernatants (lysates) were separated on 10% SDS-PAGE gel and transferred to PVDF membranes for immunoblotting. The membranes were blocked with 5% skim milk for 90 min and incubated with primary antibodies, including rabbit anti-pS129 α-synuclein (1:1000; cat. no. ab51253; Abcam), mouse anti-α-synuclein (1:1000; cat. no. 610786; BD), cleaved caspase-9 (1:1000; cat. no. 9508; CST), cleaved caspase-3 (1:1000; cat. no. 9661; CST), mouse anti-GFP (1:1000; cat. no.AG281; Beyotime Institute of Biotechnology), mouse anti-HA (1:1000; cat. no. 3724; Cell Signaling Technology), rabbit anti-TH (1:1000; cat. no. AF2185; Beyotime Institute of Biotechnology), mouse anti-β-actin (1:5000; cat. no. TA811000; OriGene Technologies, Rockville, MD, USA), and mouse anti-GAPDH (1:5000; cat. no. 60004-lg; Proteintech, Wuhan, China), overnight at 4 ℃. Subsequently, the membranes were incubated with secondary antibodies, including HRP-labeled goat anti-mouse IgG antibody (1:1000; cat. no. A0216; Beyotime Institute of Biotechnology) or HRP-labeled goat anti-rabbit IgG antibody (1:1000; cat. no. A0208; Beyotime Institute of Biotechnology), for 90 min at room temperature. The blots were developed utilizing ECL reagent (Beyotime Institute of Biotechnology) and imaged using a gel imager (Bio-Rad Laboratories, Inc.). The gray values for the bands were scanned and analyzed using ImageJ software.

### Isolation of mitochondria

Cells or fresh mouse SN tissues were dounce-homogenized in Mito buffer [20 mM Hepes (pH 7.1), 10 mM MgCl_2_, 250 mM sucrose], and centrifuged at 1000*g* for 5 min at 4 ℃. The supernatants were further centrifuged at 12,000*g* for 10 min at 4 °C, the resulting precipitate being the crude mitochondrial fraction. The precipitate was further washed with wash buffer and centrifuged at 12,000*g* for 10 min at 4 ℃, the resulting precipitate being the purified mitochondrial fraction. The crude or purified mitochondrial fraction was resuspended in Mito buffer.

### Determination of mitochondrial complex I enzyme activity

The purified mitochondria isolated from SH-SY5Y cells or fresh SN tissue (4 samples from 4 mice per group) were frozen in liquid nitrogen and thawed for three times. Mitochondrial protein concentrations were determined using a BCA kit. The activity of complex I (NADH dehydrogenase) and citrate synthase was determined using a U-3900 spectrophotometer (Hitachi, Tokyo, Japan). The reaction mixture including 0.5 M NaN_3_, 10 mM NADH, and 4 μg of mitochondrial protein was incubated at 37 ℃ for 2 min, and the oxidation rate of NADH was measured at 340 nm by adding 6 mM ubiquinone, to reflect the complex I enzyme activity. Subsequently, the reaction mixture including 1 mM DTNB, 10 mM AcCoA, and 4 μg of mitochondrial protein was incubated at 37 ℃ for 2 min, and the rate of citric acid generation was measured at 412 nm by adding oxalacetic acid, to reflect the citrate synthase activity. The enzyme activity of complex I was normalized according to the activity of citrate synthase. The enzyme activity measurements were repeated for three times independently.

### Oxygen consumption measurement

The oxygen consumption of cells or tissue mitochondria was measured by Oxygraph-2k cellular respiration apparatus (Oroboros Instruments, Innsbruck, Austria).

Cells (5 × 10^6^) were suspended in TD buffer (0.137 M NaCl, 10 mM KCl, 0.7 mM Na_2_HPO_4_, 25 mM Tris–HCl, pH 7.4) and transferred to an Oxygraph-2k chamber. The level of endogenous oxygen consumption was recorded (Base). Then oligomycin (final concentration of 2.5 μg/mL, Sigma) was added to record the level of oxygen consumption after the inhibition of ATP synthase (Oligo). At last, FCCP (final concentration of 0.1 μM, Sigma) was added to determine the uncoupling, maximal oxygen consumption (FCCP). Respiratory control rate (RCR) and leakage control rate (LCR) was respectively calculated as Base/Oligo and Oligo/FCCP.

Protein concentration of crude mitochondria from fresh SN tissue (4 samples from 4 mice per group) was determined using a BCA kit. Mitochondria (80 μg), resuspended in OCR buffer (20 mM Hepes pH 7.1, 250 mM sucrose, 2 mM KH2PO4, 10 mM MgCl_2_, 1 mM ADP), were transferred to an Oxygraph-2k chamber. Malic acid, glutamic acid and succinate (final concentration of 5 mM, respectively) were added to the chamber, to measure the total oxygen consumption of mitochondrial complex I and II (C I + C II). Then oligomycin (final 2.5 μg/mL) was added to record the level of oxygen consumption uncoupled to ATP synthase (Oligo). At last, FCCP (final 0.1 μM) was added to determine the maximal oxygen consumption (FCCP). RCR and LCR were respectively calculated as (C I + C II)/Oligo, and Oligo/FCCP.

### Determination of ATP content

The cellular ATP content was measured using a luciferin/luciferase chemiluminescence ATP determination kit (ThermoFisher Scientific). Cells (1 × 10^6^) were lysed by boiling in buffer (100 mM Tris, 4 mM EDTA) for 90 s and then centrifuged at 10,000*g* for 1 min. The supernatant (after the determination of protein concentration) was mixed with the standard reaction solution, and the chemiluminescence was detected using a multiscan spectrum (ThermoFisher Scientific). The relative ATP levels were calculated based on standard curves. In addition to determining the basal ATP synthesis of cells (Base), the cells were incubated with 15 μg/mL oligomycin for 30 min at 37 ℃ before harvesting the cells, to determine the ATP synthesis after the inhibition of ATP synthase (Oligo). The proportion of ATP synthesis sensitive to oligomycin was calculated as (Base-Oligo)/Base.

### Measurement of ROS in mitochondria

The harvested cells (about 5 × 10^5^) were incubated with 500 μL 5 μM MitoSOX™ Red (mitochondrial superoxide indicator, ThermoFisher Scientific, USA) for 25 min at 37 ℃. After washed by PBS, the cells suspended in PBS were detected by Flow Cytometry (BD Biosciences, USA), setting 5000 cells to get the median fluorescence intensity (MFI) of each sample.

### Statistics

Probability (*P*) values were calculated by using SPSS 22.0 software. Unpaired student t-test was used to compare means between two groups. Comparison of means among three or four groups was performed using one-way ANOVA. First, the homogeneity of variance was tested. If equal variances were assumed, then the *P*-values were calculated using Tukey's post hoc test. Otherwise, the *P*-values were calculated using Tamhane's T2. Difference with *P* < 0.05 was considered statistically significant.

## Results

### NDI1 is expressed and localized in mitochondria after transduced to SH-SY5Y cells

**Fig. 1:**
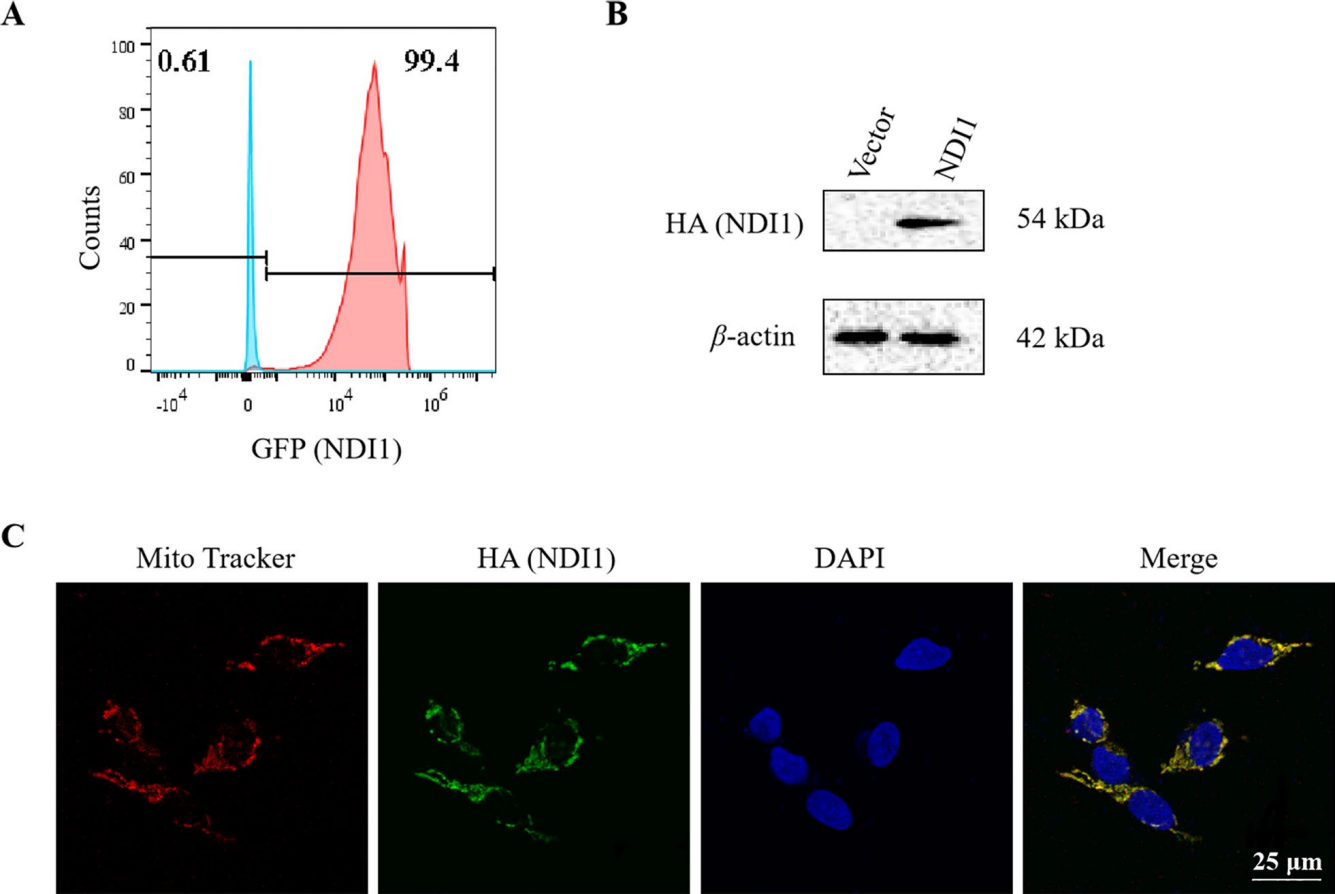
NDI1 expression and subcellular localization after transduced to SH-SY5Y cells. **A** GFP (NDI1) positive cells were analyzed by flow cytometry. **B** The expression of HA (NDI1) was examined by western blot using HA antibody with β-actin as loading control. **C** Mitochondrial localization of HA (NDI1) was verified by a confocal microscope. The LV-NDI1 transduced SH-SY5Y cells were co-stained with MitoTracker dye (red) and HA antibody (green), and counterstained with DAPI (blue). Scale bar = 25 μm

SH-SY5Y cells were transduced with concentrated LV-NDI1 at the MOI of 5. More than 99% of the cells were positively transduced, indicated by the GFP (NDI1) positive cell population determined by flow cytometry 72 h post-transduction (Fig. 1A). The expression level of HA-tagged NDI1 in the NDI1 group was detected by western blot, with vector group as the negative control (Fig. 1B). HA (NDI1) was colocalized with MitoTracker, examined by a confocal microscope (Fig. 1C). These results indicated that NDI1 protein was located in the mitochondria of SH-SY5Y cells.

### NDI1 resists changes in cell morphology, decrease in cell survival, and accumulation of pS129 *α*-synuclein in rotenone-induced PD cell model

SH-SY5Y cells transduced with LV-NDI1 were treated with rotenone, and subjected to morphological, survival, and biochemical analyses.

**Fig. 2:**
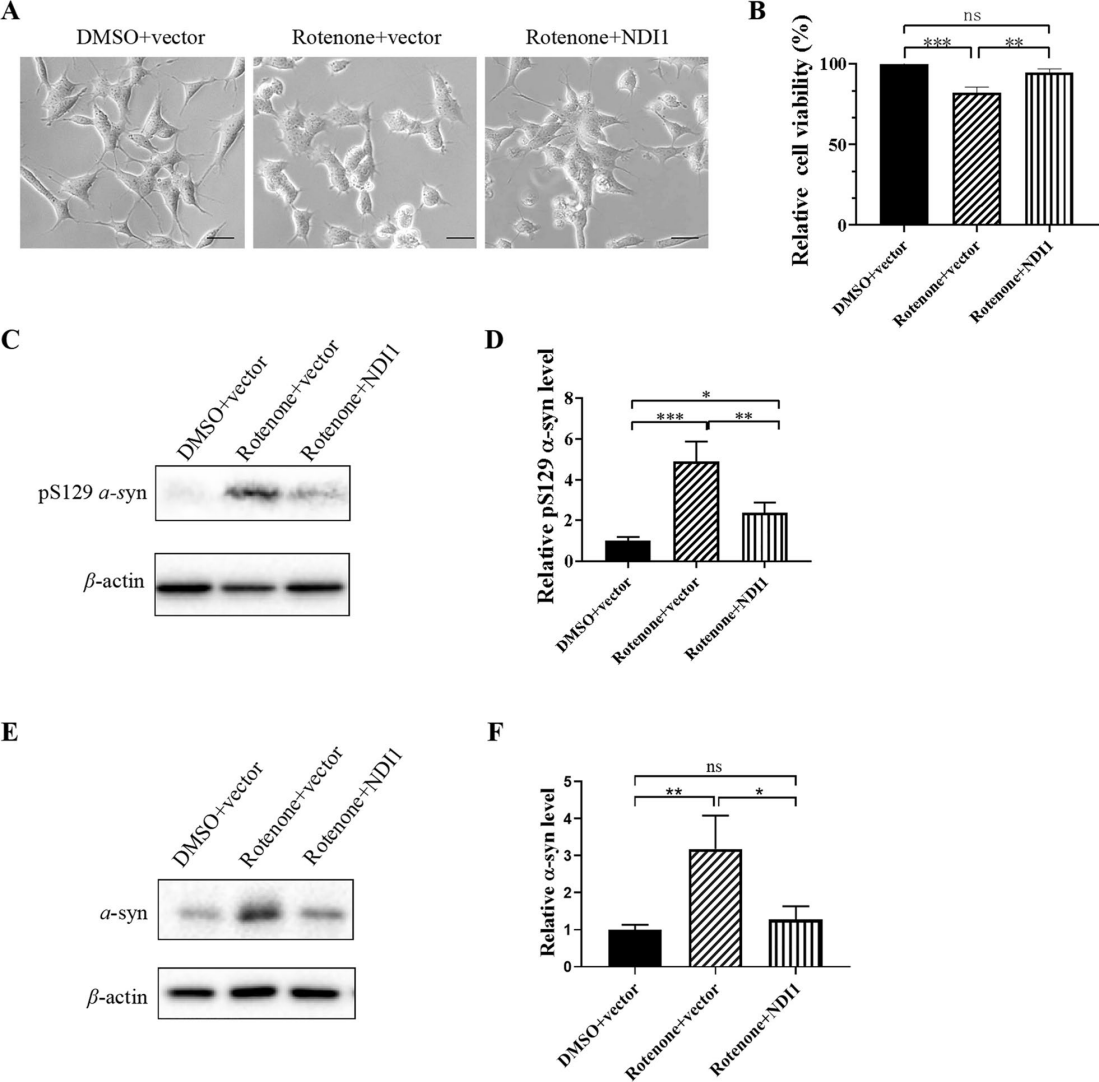
Cell morphology, survival, and the level of pS129 α-synuclein and α-synuclein protein in rotenone-induced PD cell model transduced with NDI1. **A** Changes in cell morphology were examined using an inverted phase contrast microscope. (Scale bar: 100 μm). **B** Cell viability was examined using trypan blue exclusion staining. **C**, **E** The level of pS129 *α*-synuclein, *α*-synuclein protein was detected by western blot using pS129 *α*-synuclein, *α*-synuclein antibody with β-actin as loading control. **D**, **F** Quantitative analysis of pS129 *α*-synuclein, *α*-synuclein protein level detected by western blot. Rotenone + vector group compared with DMSO + vector group, or Rotenone + NDI1 group compared with Rotenone + vector group, or Rotenone + NDI1 group compared with DMSO + vector group, *ns* not significant, *P < 0.05, **: *P* < 0.01, ***: *P* < 0.001

In retenone + vector group cells, rotenone treatment induced significant morphology changes including cell shrinkage and decrease in cell protrusions compared to DMSO + vector group cells (Fig. 2A). Notably, in retenone + NDI1 group, expression of NDI1 increased cell protrusions and cell body spreading. These results indicated that NDI1 had a therapeutic effect on the cell morphology changes induced by rotenone.

The results of trypan blue exclusion staining showed that the cell viability was significantly decreased in the rotenone + vector group compared to the DMSO + vector group (*P* < 0.001), but increased in the rotenone + NDI1 group compared to the rotenone + vector group (*P* < 0.01) (Fig. 2B). Between the DMSO + vector group and the rotenone + NDI1 group, there was no significant difference (Fig. 2B). These results indicated that NDI1 can resist almost completely the decrease in cell survival caused by rotenone.

The accumulation of pS129 *α*-synuclein in dopaminergic neurons is the basis for the formation of Lewy bodies. The level of pS129 *α*-synuclein and *α*-synuclein was detected by western blot (Fig. 2C, E). The results showed that both pS129 *α*-synuclein level and *α*-synuclein level were increased in the rotenone + vector group compared to the DMSO + vector group (*P* < 0.001, *P* < 0.01), but decreased in the rotenone + NDI1 group compared to the rotenone + vector group (*P* < 0.01, *P* < 0.05) (Fig. 2D, F).

### NDI1 restores the mitochondrial oxidative phosphorylation function in rotenone-induced PD cell model

To investigate the therapeutic effect of NDI1 on the impaired mitochondrial oxidative phosphorylation function in the rotenone-induced PD cell model, we examined the complex I enzyme activity, oxygen consumption, and ATP level.

**Fig. 3:**
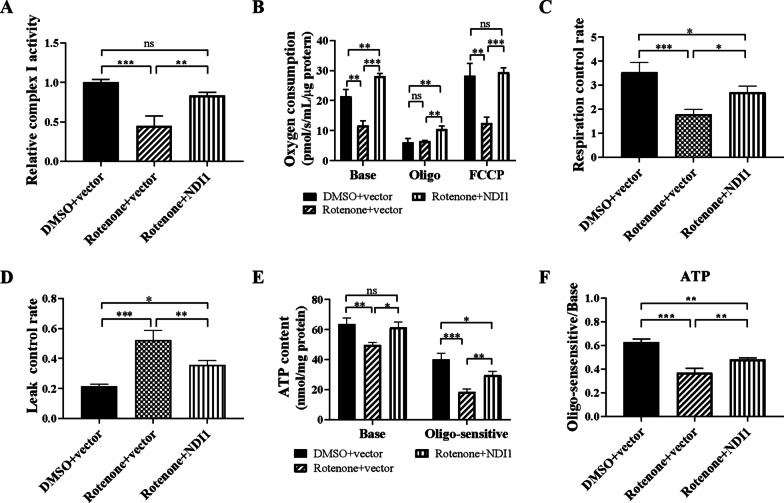
The mitochondrial oxidative phosphorylation function in rotenone-induced PD cell model transduced with NDI1. **A** The mitochondrial complex I enzyme activity was determined by measuring the NADH oxidation rate using a spectrophotometer. **B** The basal oxygen consumption (Base), ATP synthase uncoupling oxygen consumption after oligomycin treatment (Oligo), and maximum oxygen consumption after FCCP treatment (FCCP) were detected using a cellular respiration apparatus. **C** RCRs (respiratory control rates) were calculated as Base /Oligo. **D** LCRs (leakage control rates) were calculated as Oligo/FCCP. **E** The basal ATP content (Base) and oligomycin-sensitive ATP content (Oligo-sensitive) were measured using a luciferin/luciferase chemiluminescence ATP assay kit. **F** The ratios of oligomycin-sensitive ATP content were calculated as Oligo-sensitive/ Base. Rotenone + vector group compared with DMSO + vector group, or Rotenone + NDI1 group compared with Rotenone + vector group, or Rotenone + NDI1 group compared with DMSO + vector group, *ns* not significant. *P < 0.05, **P < 0.01, ***P < 0.001

NDI1 protein, an NADH dehydrogenase in mitochondria of yeast, is functionally similar to mammalian complex I, which transfers electrons from NADH into the respiratory chain. Complex I catalyzes the dehydrogenation of NADH to NAD + , whose activity can be measured by the NADH oxidation rate at 340 nm and normalized by the citrate synthase activity. The relative complex I enzyme activity was significantly decreased in the rotenone + vector group compared to the DMSO + vector group (*P* < 0.001), but restored in the rotenone + NDI1 group compared to the rotenone + vector group (*P* < 0.01) (Fig. 3A). These results showed that the activity of the endogenous mammalian complex I enzyme was reduced in response to rotenone treatment. In comparison, exogenous yeast NDI1 was resistant to rotenone and rescued rotenone-impaired complex I enzyme activity.

The oxygen consumption level was examined using Oxygraph-2k cellular respiration apparatus. First, the basal cellular oxygen consumption level was decreased in the rotenone + vector group compared to the DMSO + vector group (*P* < 0.01) but increased significantly in the rotenone + NDI1 group compared to the rotenone + vector group (*P* < 0.001) (Fig. 3B, base). Second, oxygen consumption in the presence of oligomycin was increased in the rotenone + NDI1 group compared to the rotenone + vector group (*P* < 0.01) (Fig. 3B, oligo). Oligomycin is an inhibitor of complex V (ATP synthase), uncoupling ATP synthase from the electron transport chain. Third, the maximum oxygen consumption, which was caused by uncoupling the electron transport chain using FCCP treatment, was decreased in the rotenone + vector group compared to the DMSO + vector group (*P* < 0.01) but significantly increased in the rotenone + NDI1 group compared to the rotenone + vector group (*P* < 0.001) (Fig. 3B, FCCP). In addition, mitochondrial coupling efficiency was evaluated by calculating respiratory control rate (RCR), which represents mitochondrial ATP synthase coupling, and leakage control rate (LCR), which represents proton leakage. RCR was significantly decreased in the rotenone + vector group compared to the DMSO + vector group (*P* < 0.001) but increased in the rotenone + NDI1 group compared to the rotenone + vector group (*P* < 0.05) (Fig. 3C). Conversely, LCR was significantly increased in the rotenone + vector group compared to the DMSO + vector group (*P* < 0.001) but decreased in the rotenone + NDI1 group compared to the rotenone + vector group (*P* < 0.01) (Fig. 3D). These results indicated that the basal cellular oxygen consumption level, maximum oxygen consumption level, and mitochondrial coupling efficiency were impaired in the rotenone-induced PD cell model, and these can be rescued by NDI1.

Furthermore, we examined ATP production using a luciferin/luciferase chemiluminescence ATP assay kit. The basal ATP level was decreased in the rotenone + vector group compared to the DMSO + vector group (*P* < 0.01) but increased in the rotenone + NDI1 group compared to the rotenone + vector group (*P* < 0.05) (Fig. 3E, Base). ATP production from mitochondria was measured after treatment with oligomycin, which was significantly decreased in the rotenone + vector group compared to the DMSO + vector group (*P* < 0.001) but increased in the rotenone + NDI1 group compared to the rotenone + vector group (*P* < 0.01) (Fig. 3E, oligo-sensitive). The ratio of ATP content sensitive to oligomycin was significantly decreased in the rotenone + vector group compared to the DMSO + vector group (*P* < 0.001) but increased in the rotenone + NDI1 group compared to the rotenone + vector group (*P* < 0.01) (Fig. 3F). These results showed that mitochondria-derived ATP production was decreased in the rotenone-induced PD cell model, but NDI1 can restore this mitochondrial-derived ATP level.

The complex I enzyme activity, oxygen consumption level, and ATP level were all rescued after NDI1 was transduced into rotenone-induced PD cell model, indicating that NDI1 can restore oxidative phosphorylation functions by replacing human mitochondrial complex I at the cellular level. Between the DMSO + vector group and the rotenone + NDI1 group, there was no significant difference in complex I activity (Fig. 3A), the maximum oxygen consumption (Fig. 3B) and basal ATP level (Fig. 3E), indicating that NDI1 can restore almost completely these aspects of oxidative phosphorylation functions at the cellular level.

### NDI1 resists mitochondrial ROS production and intrinsic apoptosis in rotenone-induced PD cell model

**Fig. 4:**
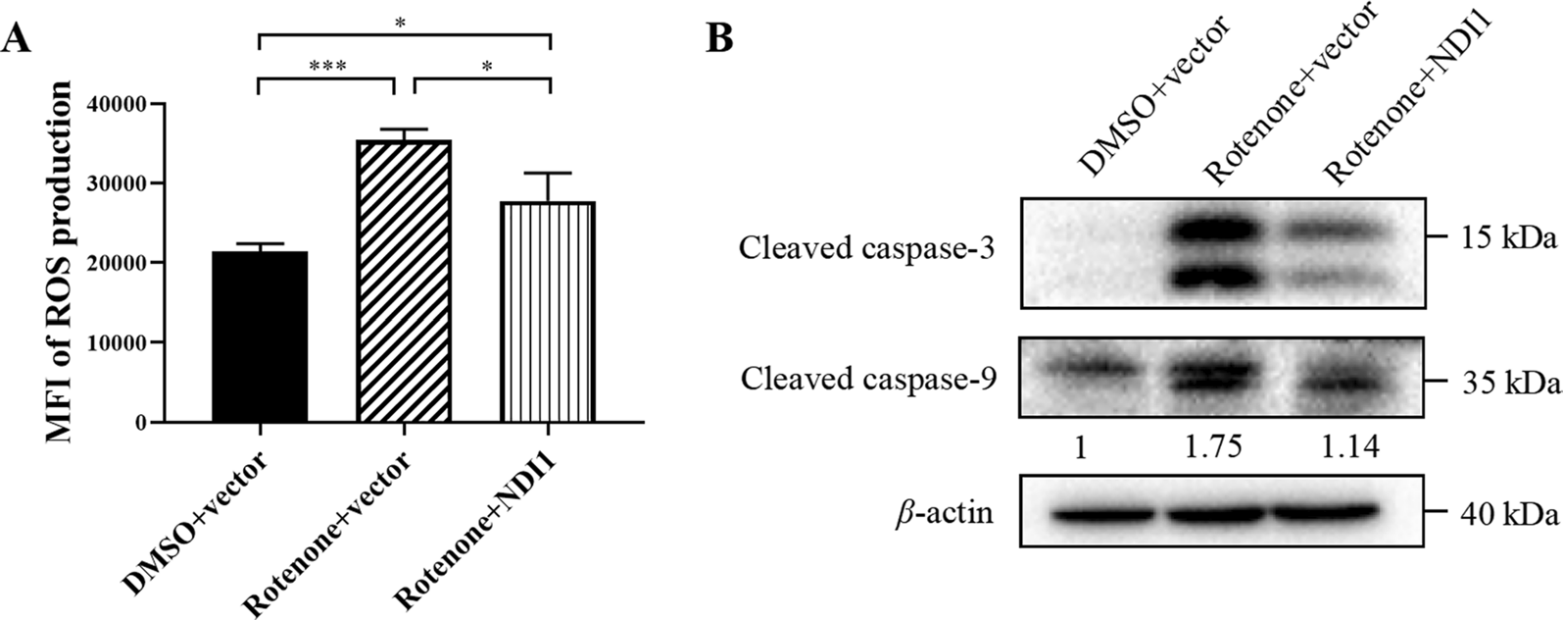
The mitochondrial ROS level and mitochondria-mediated apoptosis level in rotenone-induced PD cell model transduced with NDI1. **A** The ROS level in mitochondria was examined by MitoSOX™ staining. **B** The mitochondria-mediated apoptosis level was examined by western blot using cleaved caspase-9 antibody and cleaved caspase-3 antibody with β-actin as loading control. MFI: median fluorescence intensity. Rotenone + vector group compared with DMSO + vector group, or Rotenone + NDI1 group compared with Rotenone + vector group, or Rotenone + NDI1 group compared with DMSO + vector group, *ns* not significant. *P < 0.05, **P < 0.01, ***P < 0.001

Besides the reduced complex I activity, the increased mitochondria-derived reactive oxygen species (ROS) production, has been implicated in the pathogenesis of PD (Choong and Mochizuki 2017; Picca et al. 2021). In order to explore the level of oxidative stress after NDI1 transduction to rotenone-induced cell model, MitoSOX™, a specific dye that can recognize mitochondrial superoxide anion free radicals, was used to examine the level of ROS in mitochondria. The median fluorescence intensity (MFI) of each sample was increased in the rotenone + vector group compared to the DMSO + vector group (*P* < 0.001) but decreased in the rotenone + NDI1 group compared to the rotenone + vector group (*P* < 0.05) (Fig. 4A). The results showed that rotenone induced an increase in the oxidative stress level of the cell model, and the introduction of NDI1 could reduce the oxidative stress level.

Degeneration of the dopaminergic neurons in the SN is the cause of motor dysfunction in PD. The aberrant activation of the intrinsic apoptotic pathway may contribute to or even be a major driver of neuronal death in PD (Moujalled et al. 2021). In order to explore the level of the intrinsic (mitochondria-mediated) apoptosis after NDI1 transduction to rotenone-induced cell model, cleaved caspase-9 (activated caspase-9, initiator caspase) and cleaved caspase-3 (activated caspase-3, executioner caspase) were examined respectively. The cleaved caspase-9 and cleaved caspase-3 were increased in the rotenone + vector group compared to the DMSO + vector group, but decreased in the rotenone + NDI1 group compared to the rotenone + vector group (Fig. 4B). The results showed that rotenone induced an increase in the mitochondria-mediated apoptosis level of the cell model, and the introduction of NDI1 could reduce the mitochondria-mediated apoptosis level.

### NDI1 is efficiently expressed in dopaminergic neurons in the right substantia nigra of mice after injection of AAV5-NDI1

**Fig. 5:**
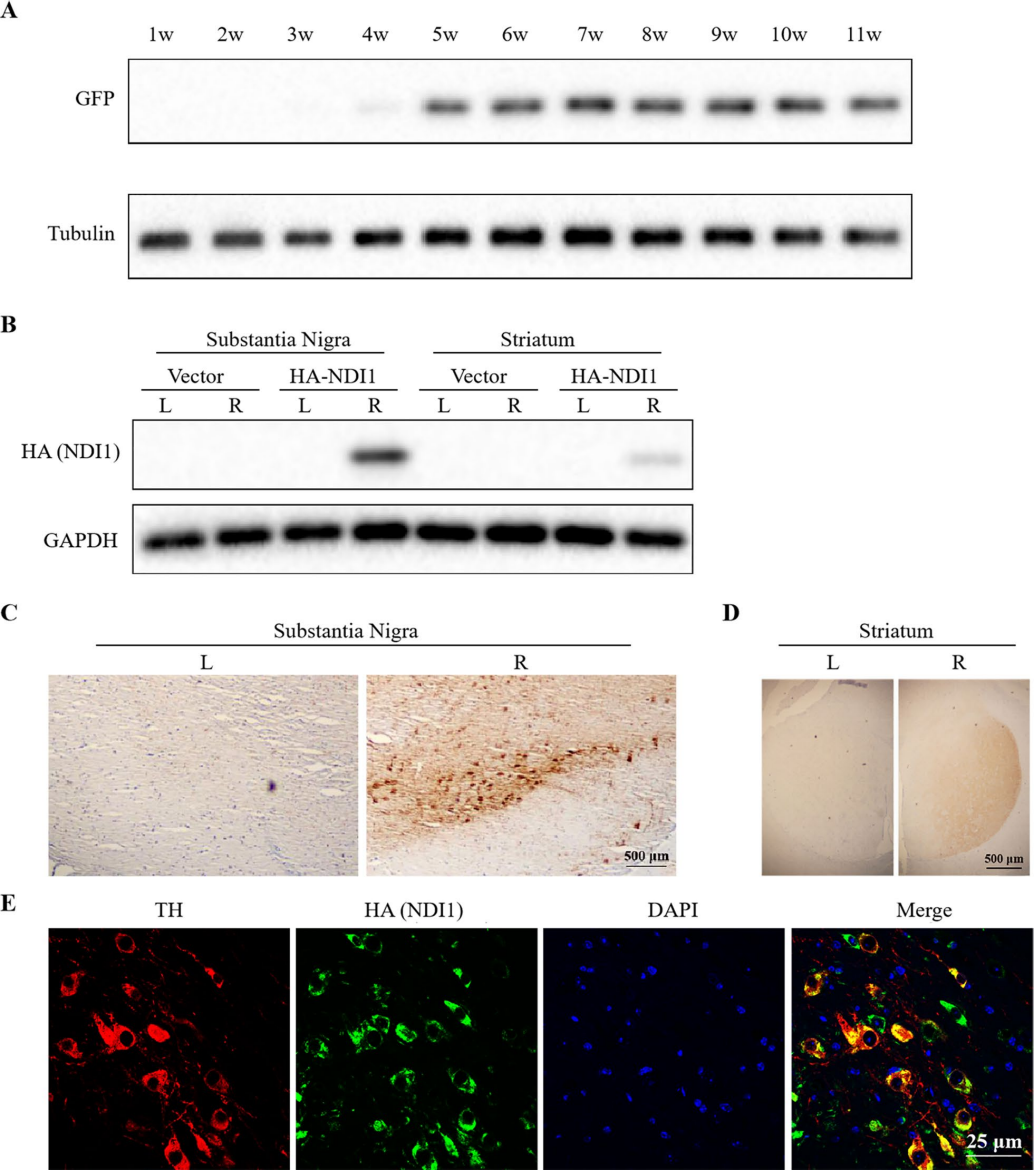
Identification of NDI1 expression in the substantia nigra and striatum of mice. **A** The time course of GFP expression in the SN of mice injected with AAV5-GFP was examined by western blot using GFP antibody with tubulin as loading control. W: week. **B** HA (NDI1) expression in the SN and striatum of mice injected with AAV5-HA-NDI1 was examined by western blot using HA antibody with GAPDH as loading control. L: left; R: right. **C**, **D** HA (NDI) expression in the SNpc or striatum of mice injected with AAV5-HA-NDI1 was examined by HA immunohistochemistry. Scale bar = 500 μm. **E** TH (tyrosine hydroxylase) and HA (NDI1) co-localization in dopaminergic neurons of SNpc was examined by a confocal microscopy. SNpc tissue section was co-stained with TH antibody (red, dopaminergic neurons) and HA antibody (green, HA), and counterstained with DAPI (blue, nucleus). Scale bar = 25 μm

The AAV genome, a single-strand DNA, must rely on the replication machinery of host cells to synthesize its complementary strand, which may delay the expression of the transduced gene. To estimate the time course of AAV5-mediated expression, we injected AAV5-GFP into the right SNpc by stereotaxic injection. The expression of GFP in the SN was detected by western blot (Fig. 5A). The results showed that low level of GFP protein was found as early as in week 4 post injection, the expression reached the highest level in week 5, and maintained its high constant level till at least week 11. Subsequently, the AAV5-NDI1 or AAV5-vector was injected unilaterally into the right SNpc of the mice. Five weeks later, western blot analysis showed that HA (NDI1) was expressed in the SN and striatum of the right brain but not in the left brain (Fig. 5B), indicating that AAV5 mediated the high expression of NDI1 in the injected lateral SN, and NDI1 reached the ipsilateral striatum through the nigrostriatal pathway although the level of NDI1 in the striatum was low. HA (NDI1) expression in the SNpc (Fig. 5C) and striatum (Fig. 5D) was also examined by immunohistochemistry. The NDI1 protein was localized in the SN and striatum of the right brain in the HA-NDI1 group. In addition, HA (NDI1) was co-localized with TH in dopaminergic neurons of SNpc examined by a confocal microscope (Fig. 5E). The results showed that NDI1 was successfully expressed in the cytoplasmic region of dopaminergic neurons. According to these data, we treated the mice with rotenone between week 5–11 post AAV5-NDI1 injection, when NDI1 would be highly expressed in the right SN.

### NDI1 injected in right substantia nigra pars compacta of rotenone-induced PD mouse model prevents decrease in the number of rotations toward right side

Injection of apomorphine, a dopamine receptor agonist, can induce equal number of rotations toward each side for healthy mice. When the nigrostriatal dopamine system in one side is injured, the dopamine receptors in the postsynaptic membrane are more sensitive to apomorphine due to innervation loss. Apomorphine dominates excitatory effect on the injured side, which makes mice rotate toward the healthy side.

**Fig. 6:**
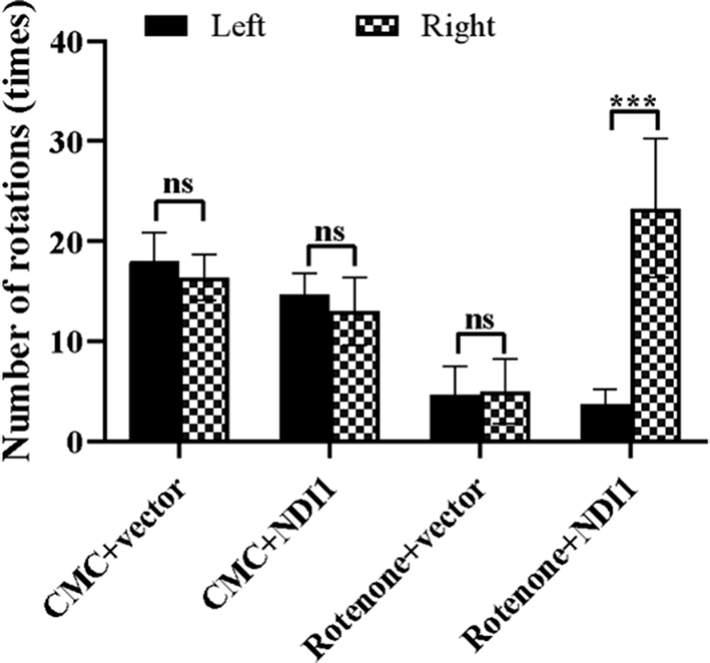
Rotation test to examine the therapeutic effect of NDI1 on ipsilateral neuropathy in rotenone-induced PD mouse model. Apomorphine induced the mice to rotate toward the healthy side. The AAV5-NDI1 was injected into the right SNpc. To compare the difference between the leftward and rightward number of rotations in each group, *ns* no significant. ***P < 0.001

The AAV5-NDI1 or AAV5-vector was injected into the right SNpc. There was no significant difference between the leftward and rightward number of rotations in the CMC + vector, CMC + NDI1, and rotenone + vector groups (Fig. 6), although the number of rotations to the left or right side was low in rotenone + vector group compared to the CMC + vector group. In contrast, the rightward number of rotations was significantly higher than the leftward number of rotations in the rotenone + ND1I group (*P* < 0.001) (Fig. 6), suggesting that the function of the right brain was better than that of the left brain, likely due to the expression of NDI1 in the injected right SNpc. These results demonstrated that NDI1 had a significant therapeutic effect on rotenone-induced neuropathy in mice.

### NDI1 protects dopaminergic neurons against neuronal toxicity in the substantia nigra of rotenone-induced PD mouse model

The main pathological feature in PD was the progressive loss of dopaminergic neurons in the SNpc, including changes in the morphology and survival of dopaminergic neurons.

**Fig. 7:**
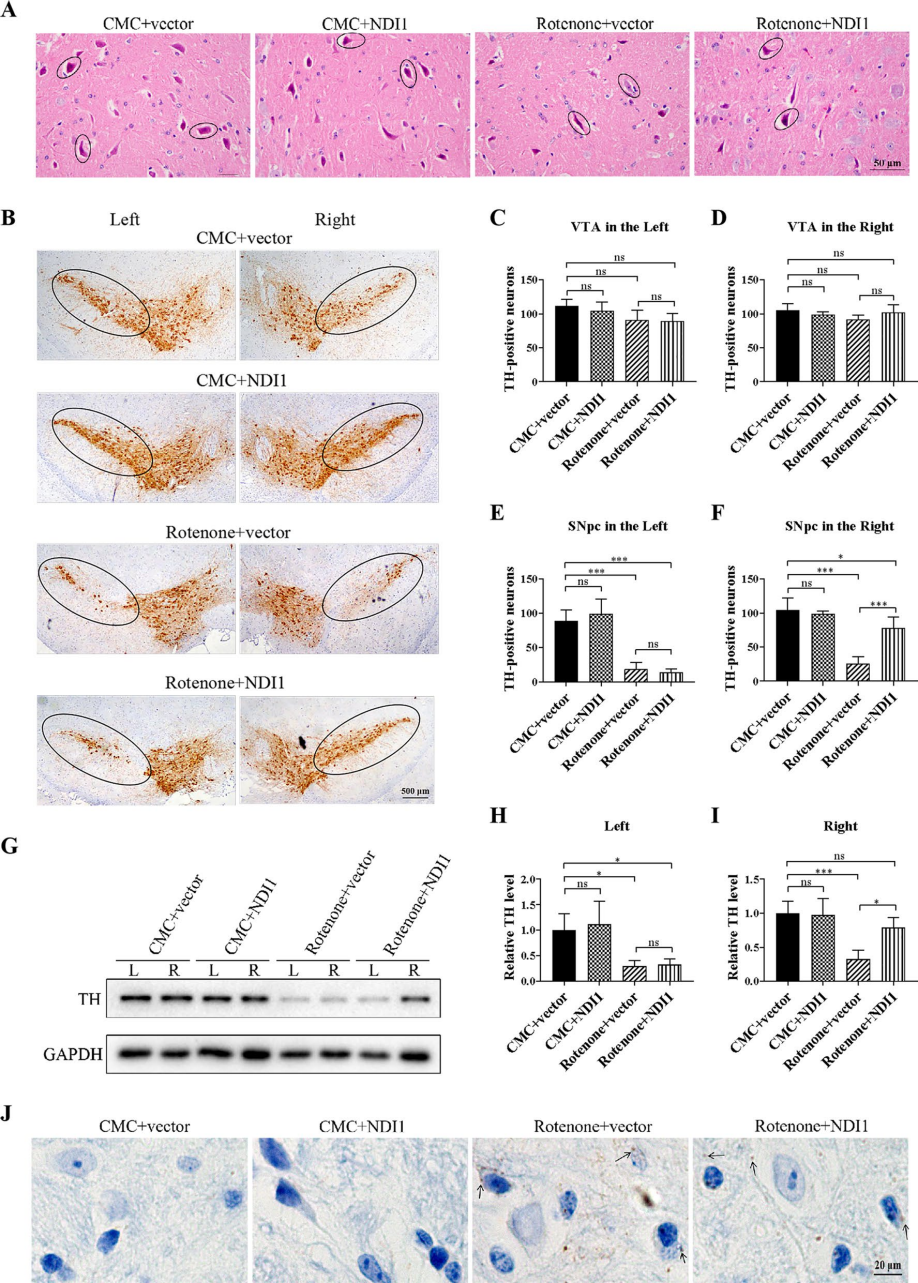
The morphology of dopaminergic neurons, number of viable dopaminergic neurons, TH protein level, and the formation of Lewy bodies in substantia nigra of rotenone-induced PD mouse model transduced with NDI1. **A** The morphology of dopaminergic neurons in the right SNpc was examined using H&E staining. Dopaminergic neurons were indicated with circles. (Scale bar: 50 μm). **B** The presence of viable dopaminergic neurons in the SNpc (indicated with circles) and VTA (out of circles) was examined using TH immunohistochemistry. Brain sections were stained with TH antibody. TH-positive cells were counted to evaluate viable dopaminergic neurons. Scale bar = 500 μm. **C**–**F** Statistical analysis of the number of TH-positive dopaminergic neurons in the left and right VTA, and in the left and the right SNpc. **G** TH protein in the SN was examined by western blot using TH antibody with GAPDH as loading control. **H**, **I** Quantitative analysis of the TH level in the left and right SN. **J** The formation of Lewy bodies in dopaminergic neurons of right SNpc was examined by pS129 *α*-synuclein immunohistochemistry. The arrow showed Lewy body (brown). Rotenone + vector group compared with CMC + vector group, or CMC + NDI1 group compared with CMC + vector group, or Rotenone + NDI1 group compared with Rotenone + vector group, or Rotenone + NDI1 group compared with CMC + vector group, *ns* not significant, *P < 0.05, ***P < 0.001. *SNpc* substantia nigra pars compacta, *VTA* ventral tegmental area, *TH* tyrosine hydroxylase

To investigate the therapeutic effect of NDI1 on the morphology of dopaminergic neurons in the rotenone-induced PD mouse model, we used H&E staining to examine the morphology of dopaminergic neurons in the right SNpc. The normal dopaminergic neurons in the CMC + vector group and CMC + NDI1 group were oval or polygonal in shape, with large and round nuclei, visible nucleoli in dark blue, and pink cytoplasm. In contrast, the residual dopaminergic neurons in the rotenone + vector group were degenerate, with nuclear pyknosis and in the shape of fibrous strips. However, in the rotenone + NDI1 group, only a small number of dopaminergic neurons was degenerated, and increasing numbers of dopaminergic neurons were in the normal oval or polygonal shape, indicating that the morphology of dopaminergic neurons partially got better (Fig. 7A). These results showed that rotenone caused severe damage to dopaminergic neurons, and NDI1 could partially protect dopaminergic neurons from rotenone-induced neuronal toxicity in the morphology.

To explore the therapeutic effect of NDI1 on the survival of dopaminergic neurons in the rotenone-induced PD mouse model, we used tyrosine hydroxylase (TH), the rate-limiting enzyme in dopamine synthesis, as a marker to evaluate the viable number of dopaminergic neurons by counting the TH-positive cells. The region of TH stained cells was in the shape of an inverted, elongated comma, the SNpc area was indicated with circles, and the VTA area was out of circles (Fig. 7B). The number of viable dopaminergic neurons in the left and right VTA (Fig. 7C, D) was not different among all groups, indicating that exposure to rotenone resulted in no obvious damage to dopaminergic neurons in the VTA. However, the number of viable dopaminergic neurons in the left and right SNpc (Fig. 7E, F) was significantly decreased in the rotenone + vector group compared to the CMC + vector group (P < 0.001), indicating that rotenone had significant damage to the left and right SNpc. In addition, the number of dopaminergic neurons in the right SNpc was significantly increased in the rotenone + NDI1 group compared to the rotenone + vector group (P < 0.001), while in the left SNpc there was no difference between these two groups (Fig. 7E, F), indicating that injection of NDI1 in the right SNpc could protect the right SNpc and resisted the damage of rotenone in the number of viable dopaminergic neurons.

In PD, the degeneration and loss of dopaminergic neurons in the SN cause a decrease in TH content. Western blot analysis was performed (Fig. 7G) to further verify the results of TH immunohistochemistry. TH level in the left and right SN (Fig. 7H, I) was decreased in the rotenone + vector group compared to the CMC + vector group (*P* < 0.05, *P* < 0.001). Notably, TH level in the right SN was increased in the rotenone + NDI1 group compared to the rotenone + vector group (*P* < 0.05), whereas there was no difference between these two groups in the left SNpc (Fig. 7H, I). Between the DMSO + vector group and the rotenone + NDI1 group, there was no significant difference (Fig. 7I). These results indicated that NDI1 protected almost completely SN against rotenone-induced decrease in TH content.

Another characteristic pathological change in PD is the formation of Lewy bodies, an eosinophilic inclusion body existing in the cytoplasm, mainly composed of *α*-synuclein and ubiquitin in the damaged dopaminergic neurons. Hyperphosphorylation at serine 129 (pS129) of *α*-synuclein induces the transformation of *α*-synuclein from monomer to oligomer, leading to the formation of Lewy bodies. Thus, pS129 *α*-synuclein was used as a marker to examine Lewy bodies (Fig. 7J). The result of pS129 *α*-synuclein immunochemistry revealed that there were more Lewy bodies present in residual neurons in rotenone + vector group compared to CMC + vector group. However, NDI1 did not reduce the number of Lewy bodies in NDI1 + rotenone group compared to rotenone + vector group.

### NDI1 restores dopamine content and decreased injury in the striatum of rotenone-induced PD mouse model

**Fig. 8:**
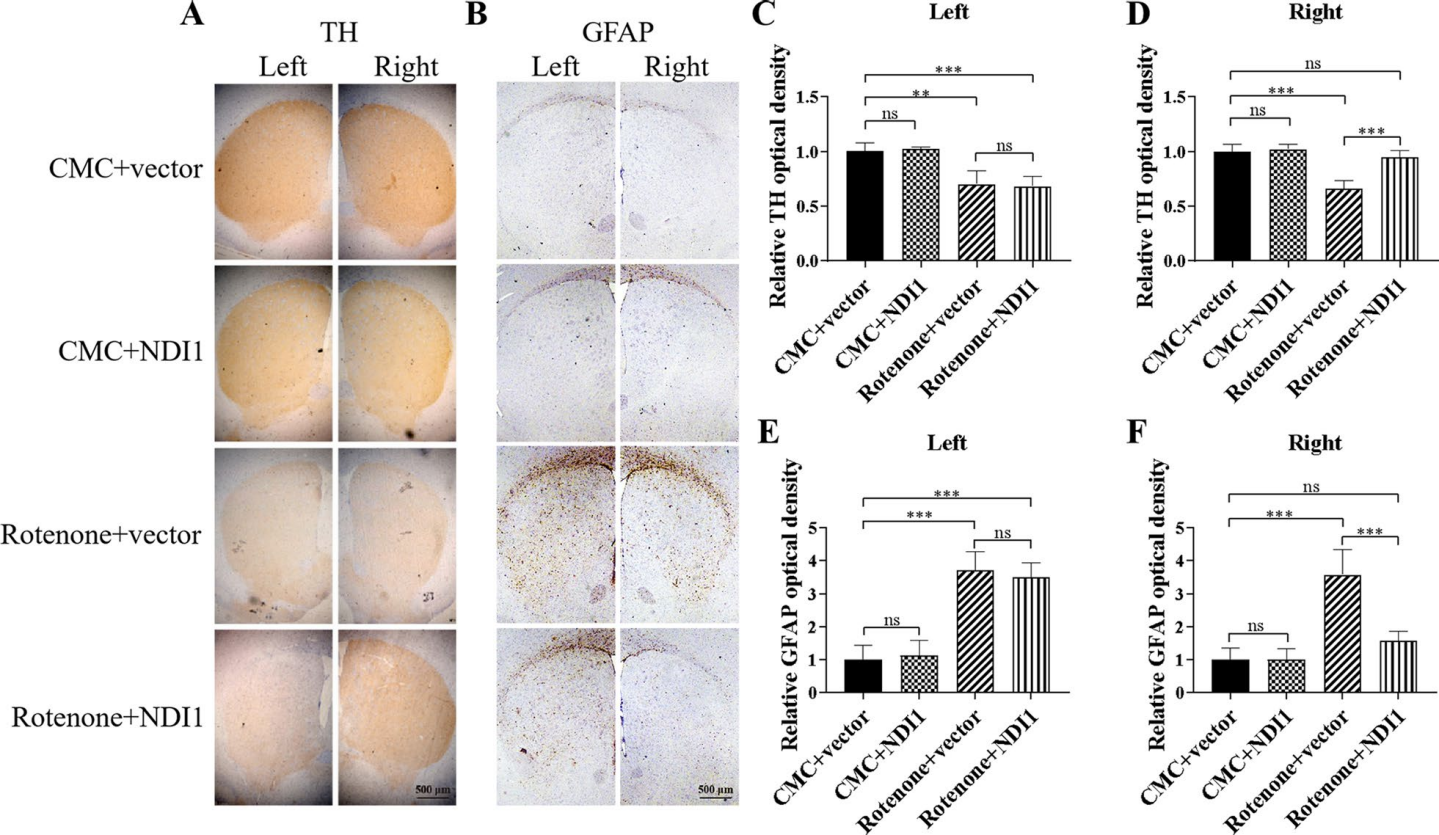
The content of TH and GFAP in striatum of rotenone-induced PD mouse model transduced with NDI1. **A** TH content in stratum (i.e. the content of dopamine) was examined by immunohistochemistry. The striatum sections were stained with TH antibody (brown). Scale bar = 500 μm. **B** GFAP (glial fibrillary acidic protein) content in stratum was examined by immunohistochemistry. The striatum sections were stained with GFAP antibody (brown). Scale bar = 500 μm. **C**, **D** TH (tyrosine hydroxylase) staining intensity was quantified by ImageJ software. The TH-positive optical density values in the left and right striatum were subjected to statistical analysis. **E**, **F** GFAP staining intensity was quantified by ImageJ software. The GFAP-positive optical density values in the left and right striatum were subjected to statistical analysis. Rotenone + vector group compared with DMSO + vector group, or Rotenone + NDI1 group compared with Rotenone + vector group, or Rotenone + NDI1 group compared with DMSO + vector group, *ns* not significant, **P < 0.01, ***P < 0.001

The content of TH in the striatum can indirectly represent the content of dopamine. TH immunohistochemical staining was performed on the striatum tissues of the mice (Fig. 8A). The intensity of TH immunohistochemical staining in striatum was quantified by ImageJ. The TH positive optical density in the left and right striatum (Fig. 8C, D) was significantly decreased in the rotenone + vector group compared to the CMC + vector group (*P* < 0.01, *P* < 0.001), suggesting that rotenone decreased the content of dopamine in the left and right striatum. Notably, the TH positive optical density in the right striatum was increased significantly in the rotenone + NDI1 group compared to the rotenone + vector group (*P* < 0.001), while there was no difference in TH staining between these two groups in the left striatum (Fig. 8C, D). Between the DMSO + vector group and the rotenone + NDI1 group, there was no significant difference (Fig. 8D). This result suggests that NDI1 can restore almost completely the content of dopamine in the striatum of rotenone-induced PD mice.

GFAP (glial fibrillary acidic protein) is an injury marker of central nervous system. GFAP immunohistochemical staining was performed on the striatum tissues of the mice (Fig. 8B). The GFAP positive optical density in the left and right striatum (Fig. 8E, F) was increased in the rotenone + vector group compared to the CMC + vector group (P < 0.001). Notably, the GFAP positive optical density in the right striatum was decreased in the rotenone + NDI1 group compared to the rotenone + vector group (*P* < 0.001), whereas there was no difference in GFAP staining between these two groups in the left striatum (Fig. 8E, F).

### NDI1 restores mitochondrial oxidative phosphorylation function in the substantia nigra of rotenone-induced PD mouse model

To investigate the effect of NDI1 on mitochondrial oxidative phosphorylation in the rotenone-induced PD mouse model, we examined the complex I enzyme activity and oxygen consumption level.

**Fig. 9:**
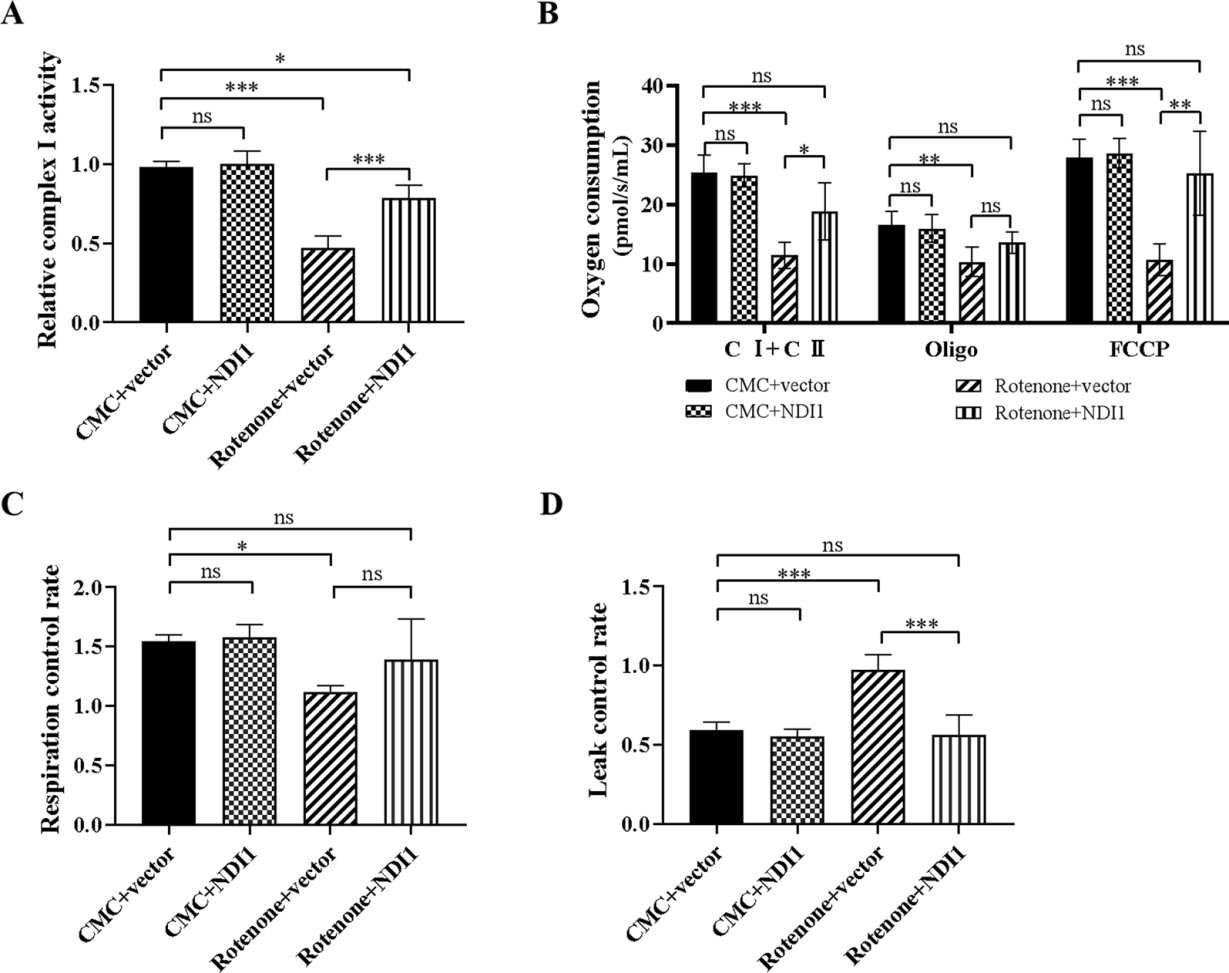
The mitochondrial oxidative phosphorylation function in right substantia nigra of rotenone-induced PD mouse model transduced with NDI1. **A** The mitochondrial complex I enzyme activity was determined by measuring the NADH oxidation rate using a spectrophotometer. **B** The total oxygen consumption of mitochondrial complexes I and II (C I + C II), ATP synthase uncoupling oxygen consumption after oligomycin treatment (Oligo), and maximum oxygen consumption after FCCP treatment (FCCP) were detected using a cellular respiration apparatus. **C** RCRs (respiratory control rates) were calculated as (C I + C II)/Oligo. **D** LCRs (leakage control rates) were calculated as Oligo/FCCP. Rotenone + vector group compared with DMSO + vector group, or Rotenone + NDI1 group compared with Rotenone + vector group, or Rotenone + NDI1 group compared with DMSO + vector group, *ns* not significant, *P < 0.05, **P < 0.01, ***P < 0.001

The activity of complex I was measured by the NADH oxidation rate and normalized by the citrate synthase activity. The relative complex I enzyme activity was significantly decreased in the rotenone + vector group compared to the DMSO + vector group (*P* < 0.001), but significantly restored in the rotenone + NDI1 group compared to the rotenone + vector group (*P* < 0.001) (Fig. 9A). These results showed that the activity of the endogenous mammalian complex I enzyme in SN was reduced in response to rotenone treatment. In comparison, exogenous yeast NDI1 was resistant to rotenone and rescued rotenone-impaired complex I enzyme activity.

Complex I + complex II (C I + C II) represented the total oxygen consumption of mitochondrial complexes I and II (Fig. 9B, C I + C II). Oxygen consumption of C I + C II, after oligomycin treatment (Fig. 9B, oligo), and after FCCP treatment (Fig. 9B, FCCP) were decreased in the rotenone + vector group compared to the CMC + vector group (*P* < 0.001, *P* < 0.01, *P* < 0.001). Notably, Oxygen consumption of C I + C II and FCCP in the rotenone + NDI1 group were increased compared to the rotenone + vector group (*P* < 0.05, *P* < 0.01).

RCR (respiratory control rate) was decreased in the rotenone + vector group compared to the CMC + vector group (*P* < 0.05) (Fig. 9C). LCR (leakage control rate) was increased significantly in the rotenone + vector group compared to the CMC + vector group (*P* < 0.001). Notably, LCR was significantly decreased in the rotenone + NDI1 group compared to the rotenone + vector group (*P* < 0.001) (Fig. 9D). Between the DMSO + vector group and the rotenone + NDI1 group, there was no significant difference in the oxygen consumption of total, after oligomycin or FCCP treatment (Fig. 9B), and in RCR and LCR (Fig. 9C, D). These results indicated that the total oxygen consumption of mitochondrial complexes I and II, the oxygen consumption of uncoupling ATP synthase, the oxygen consumption of uncoupled maximal respiration, and the mitochondrial coupling efficiency were impaired in the rotenone-induced PD mouse model, and NDI1 can rescue the above impaired respiration function to approximately normal level.

## Discussion

In this study, rotenone was used to establish the PD cell model. The results show that rotenone induces significant changes in cell morphology, cell survival, *α*-synuclein and pS129 *α*-synuclein accumulation, mitochondrial oxidative phosphorylation, oxidative stress and mitochondria-mediated apoptosis (Figs. 2, 3, 4). Rotenone may lead to *α*-synuclein accumulation in the following two ways: (i) increasing the expression of *α*-synuclein protein by enhancing the transcription of the *α*-synuclein gene and (ii) preventing the degradation of *α*-synuclein protein through the ubiquitin–proteasome system, and meanwhile increasing phosphorylation of *α*-synuclein at serine 129, which makes it more prone to aggregation (Cookson and Brug 2008). The aberrant activation of the intrinsic apoptotic pathway may contribute to or even be a major driver of neuronal death in PD (Moujalled et al. 2021). Our study in rotenone-induced PD cell model showed that the level of cleaved caspase-9 and cleaved caspase-3 were increased, which indicates that rotenone induced an increase in the mitochondria-mediated apoptosis level, similar as the conclusion reported (Guo et al. 2022). Some studies have used rotenone to establish PD mouse model (Alam and Schmidt 2002; Sherer et al. 2003; Johnson and Bobrovskaya 2015). Rotenone is a lipophilic compound, which can easily cross the blood–brain barrier. Rotenone inhibits the mitochondrial complex I in the brain. However, it only leads to the degeneration of dopaminergic neurons in the SNpc, suggesting that dopaminergic neurons in the SN were susceptible to rotenone. In this study, rotenone was used to generate the PD mouse model. The results show that rotenone induces significant neuropathological changes and decreased mitochondrial oxidative phosphorylation in SN (Figs. 7, 8, 9). Our study shows that the activity of yeast NDI1 is not affected by rotenone treatment (Figs. 3A, 9A), demonstrating the efficacy of NDI1 gene expression (Figs. 1B, 5B), and further supporting yeast NDI1 is a rotenone-resistant NADH dehydrogenase (Melo et al. 2004).

In this study, the fusion gene composed of NDI1 gene and mitochondrial targeting sequence was integrated into the nuclear genome. The fusion gene was expressed in the cytoplasm, then the NDI1 protein was transferred into the mitochondria. Some studies demonstrated that the expression of yeast NDI1 in rats did not induce an immune response, which may be due to that the foreign protein is localized in the mitochondria matrix and can evade immune monitoring (Marella et al. 2011).

The adeno-associated virus (AAV) is a non-enveloped DNA virus, and belongs to the parvoviral family with linear single-strand DNA genome of about 4.7 kb. AAV has the advantages such as the safety with low immunogenicity, long-term stable expression for the transduced gene, the ability to infect dividing and non-dividing cells including neurons in vivo (Balakrishnan and Jayandharan 2014). However, the disadvantages of AAV include low loading capacity for the recombinant gene and low infection efficiency to rapidly dividing cells in vitro (Balakrishnan and Jayandharan 2014). Recombinant AAV DNA generally remains in the nuclei of the transduced cells in non-integrated form, leading to the lower expression of the recombinant gene in progeny cells (Balakrishnan and Jayandharan 2014). Therefore, in this study, SH-SY5Y cells were transduced with recombinant lentivirus (LV-NDI1) instead of recombinant adeno-associated virus (AAV-NDI1).

AAV has been widely utilized as a vector for gene therapy in neural tissues (LeWitt et al. 2011; Blits and Petry 2016; Burger et al. 2005; Singh and Sen 2016; Bradbury et al. 2020; Chen et al. 2021). Clinical trials have demonstrated that AAV-mediated gene therapy for PD is with promising safety, efficacy and persistency (Christine et al. 2019; Deverman et al. 2018; Qu et al. 2019; Piguet et al. 2017). Currently, AAV2 is the preferred serotype of AAV in phase I and II clinical trials of gene therapy for PD, and rAAV2 is usually delivered through stereotactic injection into the brain (Blits and Petry 2016). No severe side effects have been reported in these clinical trials of gene therapy (Blits and Petry 2016). For example, the phase I clinical trial of injecting AAV2-GAD into the brain of PD patients confirmed the safety and expression stability of GAD (Kaplitt et al. 2007). In our study, an AAV5 vector was used. AAV5 has higher infection efficiency and spreads more widely in SN compared to AAV2 (Weinberg et al. 2013; Markakis et al. 2010; Pajarillo et al. 2020). To test the safety of NDI1 gene therapy, we set up a control group (CMC + NDI1 group), in which AAV5-NDI1 was injected into normal mice. The results (Figs. 5, 7, 8, 9) showed that AAV5-NDI1 was highly expressed in dopaminergic neurons after transduction into the right SNpc, and that there was no significant difference between the CMC + NDI1 group and the CMC + vector group, indicating the safety of NDI1 gene therapy.

Currently, except traditional drugs, there are three main therapeutic strategies, for mitochondrial respiratory chain complex I deficiency. One is the mitochondrial transplantation therapy. Chang et al. transferred mitochondria into fibroblasts derived from patients with MERRF syndrome (Chang et al. 2013). However, the transplantation efficiency was low, and the mitochondria could not be maintained for a long time. The other one is using cell permeable complex I protein, but it was unable to maintain a stable level for a long term (Pepe et al. 2014). In addition, the preparation, purification, and stable preservation of protein were still far from the requirement of clinical therapy. The third one employs the complex I gene therapy. The preclinical study of ND4 gene therapy for Leber hereditary optic neuropathy had been completed (Yu et al. 2012), and the clinical trials of ND4 gene therapy have been conducted (Koilkonda et al. 2014). This strategy only aims at the functional defect of a single subunit of the human complex I. Previous studies demonstrated that the yeast complex I, composed of a single subunit (NDI1 protein), can homologously replace the mammalian complex I, which consists of 45 subunits (Santidrian et al. 2013; Bordt et al. 2017). Walker et al. reported that the yeast NDI1 gene was transduced into the Drosophila model with deficiency of complex I assembly factor. The results showed that the defective phenotype of drosophila complex I was partially or completely rescued, indicating that the yeast NDI1 can replace defective drosophila complex I (Cho et al. 2012). The cell permeable TAT-NDI1 protein prepared in vitro was injected into the peritoneum of the rat model with heart disease. Cardiomyocyte mitochondria extracted from the heart disease rat model which injected with cell permeable TAT-NDI1 protein displayed increase of complex I function and recovery in rat myocardial infarction, indicating that the yeast NDI1 can replace defective rat complex I (Mentzer et al. 2014). In lung cancer cells with impaired mitochondrial oxidative phosphorylation after removing microfilament binding protein fascin, yeast NDI1 can restore its mitochondrial respiratory function (Lin et al. 2019). Using the NDI1 gene to target the defective complex I could solve the problem of axon damage and neuron loss in Multiple Sclerosis disease model (Talla et al. 2020). Our gene therapy results in vitro showed that NDI1 resisted the change of cell morphology, the decrease of cell survival and the accumulation of pS129 *α*-synuclein induced by rotenone (Fig. 2). NDI1 significantly increased the level of oxygen consumption, mitochondrial coupling efficiency, the level of ATP, and especially complex I activity in PD cell model (Fig. 3). NDI1 resists mitochondrial ROS production and intrinsic apoptosis in PD cell model (Fig. 4). Our results of NDI1 gene therapy in vivo showed that the rotation number to the right side (therapy side) was significantly increased (Fig. 6). And the number of viable dopaminergic neurons in SNpc, the expression of TH in SN, the content of dopamine in the striatum, oxygen consumption and mitochondrial coupling efficiency in SN, especially complex I activity in SN, in the right side (therapy side) of PD mouse model, were significantly increased (Figs. 7I, 8D, 9). In this study, yeast NDI1 significantly decreased pS129 *α*-synuclein level in the rotenone-induced PD cell model (Fig. 2D). However, yeast NDI1 did not prevent the formation of Lewy bodies in the right SNpc (Fig. 7J), which requires future study to explain. Furthermore, this study showed that rotenone had no damage to dopaminergic neurons in VTA (Fig. 7B–D), while had damage to those in SNpc. Several studies have indicated that the death of dopaminergic neurons in SN may be related to the activation of K_ATP_ channels (Liss et al. 2005). K_ATP_ channels are hetero-octamers, formed with four regulatory sulphonylurea receptor (SUR) subunits (SUR1, SUR2A, or SUR2B) and four inwardly rectifying potassium channel subunits (Kir6.1 or Kir6.2). In the rotenone-induced PD mouse model, the SUR1 mRNA expression level was two-fold higher in dopaminergic neurons of SN than in those of VTA, suggesting that the selective up-regulation of SUR1 leads to the damage in dopaminergic neurons of SN (Han et al. 2018).

## Conclusion

Yeast NDI1 can rescue the defects of electron transport and oxidative phosphorylation of mitochondrial complex I in rotenone-induced PD cell and mouse models, and can ameliorate rotenone-induced neurobehavioral impairment and neuropathological damages on dopaminergic neurons in the SNpc. This study may provide a basis for yeast NDI1 gene therapy for mitochondrial diseases caused by complex I defects, such as sporadic PD.

## Data Availability

The datasets used and analyzed during the current study are available from the corresponding authors on reasonable request.

## Abbreviations

rAAV: Recombinant adeno-associated virus
AAV: Adeno-associated virus
ATCC: American Type Culture Collection
ATP: Adenosine triphosphate
AADC: Aromatic l-amino acid decarboxylase
BCA: Bicinchoninic acid
C I: Complex I
C II: Complex II
CMC: Carboxymethyl cellulose
DMEM: Dulbecco's modified Eagle's medium
ECL: Enhanced chemiluminescence
ES: Embryonic stem
FBS: Fetal bovine serum
FCCP: Carbonyl cyanide-*p*-trifluoromethoxyphenylhydrazone
GAPDH: Glyceraldehyde-3-phosphate dehydrogenase
GCH: Guanosine triphosphate cyclohydrolase
GFAP: Glial fibrillary acidic protein
GFP: Green fluorescent protein
H&E: Hematoxylin–eosin
HRP: Horseradish peroxidase
LCR: Leakage control rate
LV: Lentivirus
IHC: Immunohistochemistry
iPS: Induced pluripotent stem
MERRF: Myoclonus epilepsy associated with ragged-red fibers
MFI: Median fluorescence intensity
mtDNA: Mitochondrial DNA
NADH: Nicotinamide adenine dinucleotide
NDI1: NADH-quinone oxidoreductase
OCR: Oxygen consumption rate
Oligo: Oligomycin
PD: Parkinson's disease
RCR: Respiratory control rate
SN: Substantia nigra
SNpc: Substantia nigra pars compacta
SUR: Sulphonylurea receptor
TH: Tyrosine hydroxylase
VTA: Ventral tegmental area
